# Does Temperature Modify the Effects of Rain and Snow Precipitation on Road Traffic Injuries?

**DOI:** 10.2188/jea.JE20140244

**Published:** 2015-08-05

**Authors:** Won-Kyung Lee, Hye-Ah Lee, Seung-sik Hwang, Ho Kim, Youn-Hee Lim, Yun-Chul Hong, Eun-Hee Ha, Hyesook Park

**Affiliations:** 1 Department of Social and Preventive Medicine, Inha University School of Medicine, Incheon, Republic of Korea; 2 Department of Preventive Medicine, School of Medicine, Ewha Womans University, Seoul, Republic of Korea; 3 Department of Epidemiology and Biostatistics, Seoul National University, Seoul, Republic of Korea; 4 Institute of Environmental Medicine, Seoul National University of Medical Research Center, Seoul National University, Seoul, Republic of Korea; 5 Department of Preventive Medicine, School of Medicine, Seoul National University, Seoul, Republic of Korea

**Keywords:** injuries, precipitation, snow, traffic accidents, weather

## Abstract

**Background:**

There are few data on the interaction between temperature and snow and rain precipitation, although they could interact in their effects on road traffic injuries.

**Methods:**

The integrated database of the Korea Road Traffic Authority was used to calculate the daily frequency of road traffic injuries in Seoul. Weather data included rain and snow precipitation, temperature, pressure, and fog from May 2007 to December 2011. Precipitation of rain and snow were divided into nine and six temperature range categories, respectively. The interactive effects of temperature and rain and snow precipitation on road traffic injuries were analyzed using a generalized additive model with a Poisson distribution.

**Results:**

The risk of road traffic injuries during snow increased when the temperature was below freezing. Road traffic injuries increased by 6.6% when it was snowing and above 0°C, whereas they increased by 15% when it was snowing and at or below 0°C. In terms of heavy rain precipitation, moderate temperatures were related to an increased prevalence of injuries. When the temperature was 0–20°C, we found a 12% increase in road traffic injuries, whereas it increased by 8.5% and 6.8% when it was <0°C and >20°C, respectively. The interactive effect was consistent across the traffic accident subtypes.

**Conclusions:**

The effect of adverse weather conditions on road traffic injuries differed depending on the temperature. More road traffic injuries were related to rain precipitation when the temperature was moderate and to snow when it was below freezing.

## INTRODUCTION

Injuries, particularly those related to road traffic accidents, are the leading cause of death among young people and remain an important public health problem. In total, 1.24 million people die worldwide from traffic accidents each year.^1^ Some experts have reported that the disability-adjusted life years (DALYs) attributable to road traffic injuries increased from 57 million in 1990 to 75 million in 2010, an increase of 33.2%, and that road traffic injuries increased from the 12th to the 10th leading cause of deaths during the same period.^2^^,^^3^ In an effort to reduce the increasing trend in road traffic fatalities and to save an estimated 5 million lives during the decade, the United Nations announced “the Decade of Action for Road Traffic Safety 2011–2020” in 2010.^4^^,^^5^

Many researchers have examined the relationship between adverse weather conditions and road traffic injuries. Since the early 1980s, studies have evaluated the effects of rain or snow precipitation on traffic accidents using matched-pair analyses^5^^–^^8^ and regression and time series analyses.^9^^–^^13^ Despite some inconsistencies, most studies have found that rain and snow precipitation contribute to increased risk of traffic accidents and road traffic injuries.^14^^,^^15^ Recent studies have found a negative lagged effect of precipitation, differences between the increase in the risk according to police and insurance data, and different effects according to the severity of the traffic accident and the age and gender of drivers.^12^^,^^16^^,^^17^ However, research on the effect of the interaction between temperature and rain or snow precipitation on road traffic injuries is lacking.

It is important to evaluate the combined effects of temperature and adverse weather conditions (ie, snow or rain precipitation) on road traffic injuries to develop more effective preventative measures. Thus, this study explored changes in the risk associated with snow or rain precipitation for road traffic injuries as a function of changes in temperature. We also evaluated whether this relationship differed according to subtypes of road traffic injury.

## METHODS

### Data

Seoul, the largest city in Korea, is located at 37°34′ N and 126°58′ E and has a temperate climate. Meteorological data for Seoul were obtained from the Korea Meteorological Administration^18^ and included temperature (°C), humidity (%), barometric pressure (hPa), rain precipitation (mm), snow precipitation (cm), and fog from one meteorological observatory located in Jongro-gu, in the center of Seoul. Temperature and barometric pressure were summarized as mean daily values. The amount of precipitation was analyzed in terms of the daily accumulation. Days with rain precipitation were categorized into light and heavy according to the median value of 3.5 mm. Snow precipitation was analyzed in terms of its mean daily depth.

There are 19 710 776 registered vehicles in Korea, which has a population of 48 183 586. Data regarding road traffic injuries were obtained from the integrated traffic accident database, maintained by the Korea Road Traffic Authority (KoRoad).^19^ The database includes data on traffic accidents supplied by police, insurance companies, and mutual aid associations on all motor vehicles, including buses, taxis, and heavy trucks. We calculated the daily frequency of road traffic injuries in Seoul from May 2007 to December 2011. Traffic accidents resulting in property damage alone were excluded. Road traffic injuries were categorized by the severity of injuries, the gender and age of the party responsible, and the type of accident. Severe injuries were defined as those requiring more than 3 weeks of medical treatment, and all others were defined as mild injuries. Individuals were classified into two groups: those under 65 years of age and those 65 years of age and older. Accidents were divided into two types: car-to-car and car-to-pedestrian accidents. The study was approved by Korea University Institutional Review Board (1040548-KU-IRB-13-164-A-1) in December 2013.

### Analysis

The meteorological characteristics of each season were summarized. Spring, summer, fall, and winter were defined as March–May, June–August, September–November, and December–February, respectively, according to the meteorological definitions for the northern hemisphere.

The interactive effects of temperature and wet road conditions on road traffic injuries were evaluated with a generalized additive model (GAM) with a Poisson distribution. First, we explored the separate effects of adverse weather conditions. Second, temperature was categorized according to range and distribution of the mean daily temperatures on days with rain and/or snow precipitation. An interaction term consisting of rain and/or snow precipitation with temperature category was included in the model. When appropriate, the combined effects of rain and snow precipitation were evaluated by subgroup according to category of mean daily temperature. To control for confounders, the final model considered barometric pressure, fog, and calendar-related variables, such as day of the week and holidays. We adjusted for the effect of snow precipitation when the influence of rain precipitation was evaluated, and vice versa.

$$\begin{array}{l} \text{Log}(\text{E(Y)})=\beta_{0}+factor(precipitationwithtemperature)\\ +factor(snow)+factor(fog)\\ +factor(dayoftheweek)+factor(holiday)\\ +S_{1}(pressure)+S_{2}(date) \end{array}$$

Daily counts of road traffic injuries had the expected value of Y, which was derived from the covariates. Categories of rain and/or snow precipitation with temperature were evaluated in the models using a smoothing spline (S) for date; there were six degrees of freedom (df) for each year and 30 df during the study period. The results are expressed as percentage changes.

Statistical analyses were performed using the SAS (ver. 9.3; SAS Inc., Cary, NC, USA) and ‘R’ (ver. 2.15.0; R Foundation for Statistical Computing, Vienna, Austria) software with the “mgcv” and “splines” packages.

## RESULTS

**Figure 1.**
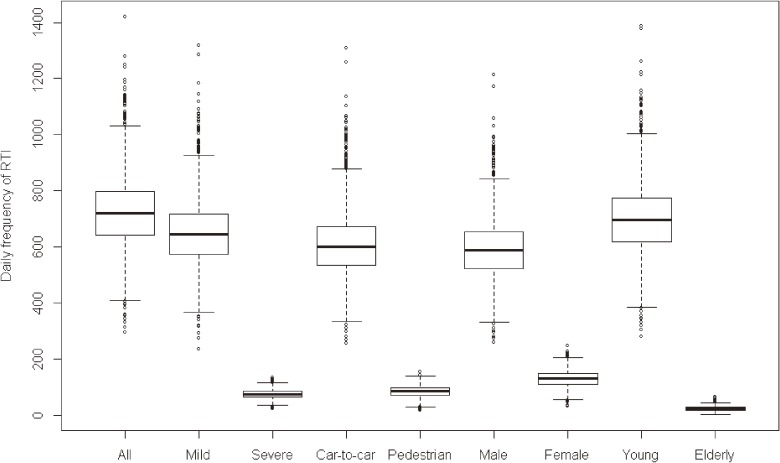
Daily frequency of road traffic injuries in Seoul from May 2007 to December 2011. “All” means all road traffic injuries (RTI); “Severe” means severe RTI which required ≥3 weeks of medical treatment, while “Mild” means mild RTI which required less than 3 weeks of treatment; “Car-to-car” is RTI from car-to-car accidents, while “Pedestrian” is RTI from car-to-pedestrian accidents; “Male” means RTI induced by male drivers, while “Female” means RTI induced by female drivers; “Young” is RTI induced by drivers younger than 65 years of age, while “Elderly” is RTI induced by the drivers aged 65 years and older.

From May 1, 2007, to December 31, 2011, the mean daily frequency of traffic accidents was 510, and the mean daily frequency of road traffic injuries was 725. The corresponding incidence of road traffic injuries was 216 per 100 000 person-months. The daily frequencies of road traffic injuries by subgroup are presented in Figure 1. The major types of road traffic injuries involved mild injuries, car-to-car accidents, and male or young drivers. Severe injuries, car-to-pedestrian accidents, accidents involving female drivers, and accidents involving elderly drivers accounted for 10.3%, 11.7%, 17.9%, and 32.5% of all road traffic injuries, respectively.

Between May 2007 and December 2011, the minimum mean daily temperature was −14.6°C, and the maximum mean daily temperature was 30°C. Precipitation was observed during 33.2% (567 days) of the study period (Table 1). Precipitation occurred during 51.3% of summer days and 22.7% of winter days. The mean daily amount of precipitation was 22 mm in the summer and 3.2 mm in the winter. Snow was observed on roads on 132 days. The average depth of snow was 4.02 cm in the winter.

**Table 1.** Meteorological characteristics of Seoul from May 2007 to December 2011

	Spring (*n* = 399)		Summer (*n* = 460)		Fall (*n* = 455)		Winter (*n* = 392)	
			
	Mean	Range	Mean	Range	Mean	Range	Mean	Range
Mean temperature (°C)	12.2	−2.13, 24.4	24.4	14.9, 30.0	14.9	−4.2, 27.0	−0.93	−14.6, 12.2
Relative humidity (%)	56.1	19.4, 94.5	71.2	24.0, 93.8	61.7	25.7, 93.0	55.1	27.0, 96.3
Pressure (hPa)	1005	984, 1020	998	987, 1009	1008	994, 1021	1013	988, 1027
Precipitation (mm)^a^	8.5	0.1, 69	22	0.1, 302	12.1	0.1, 260	3.2	0.1, 35
Snow (cm)^a^	1.4	0.04, 8.65	—	—	0.33	0.16, 0.61	4.02	0.03, 23.2
	*n*	%	*n*	%	*n*	%	*n*	%

Day with precipitation	120	30.1	236	51.3	122	26.8	89	22.7
Day with light precipitation^b^	55	13.8	89	19.3	62	13.6	68	17.3
Day with heavy precipitation^b^	65	16.3	147	32.0	60	13.2	21	5.4
Day with snow	12	3.0	—	—	5	1.1	115	29.3
Day with fog	34	8.5	55	12.0	21	4.6	17	4.3

Spring (Mar.–May), Summer (Jun.–Aug.), Fall (Sep.–Nov.), Winter (Dec.–Feb.).

^a^The amount of precipitation refers to the accumulated depth of precipitation on that day. The amount of snow refers to the mean daily depth of snow.

^b^Light precipitation was defined as less than 3.5 mm (the median), and heavy precipitation was defined as 3.5 mm or more.

Table 2 presents the separate effects of temperature, rain precipitation, and snow precipitation. The number of road traffic injuries increased during heavy rain precipitation. Road traffic injuries increased by 10.5% over the figure on dry days during days with more than 3.5 mm of rain precipitation. Cold temperatures were associated with more injuries related to traffic accidents. Injuries increased by 4.82% on days with a mean temperature below −5°C compared with days with a mean temperature above 20°C. Snow on roads was related to an 11.1% increase in the risk of injuries compared to days when there was no snow on the roads.

**Table 2.** Separate effects of temperature and wet conditions on road traffic injuries, 2007 to 2011

	All road traffic injuries			

	% change	95% CI		*P*-value
Daily mean temperature^a^				
10–20°C	0.12	−0.67	0.92	0.763
0–10°C	1.28	0.07	2.25	0.038
−5–0°C	3.08	1.53	4.64	<0.001
<−5°C	4.82	3.08	6.60	<0.001
Amount of precipitation^b^				
Light precipitation	−0.20	−0.74	0.34	0.475
Heavy precipitation	10.5	9.93	11.1	<0.001
Days between last and current precipitation^b^				
0 day	4.43	3.84	5.02	<0.001
1 day	1.25	0.32	2.18	0.008
2–3 days	3.03	2.15	3.92	<0.001
4–6 days	7.68	6.73	8.64	<0.001
≥7 days	9.28	8.13	10.4	<0.001
Snow^c^				
Snow	11.1	10.1	12.1	<0.001

CI, confidence interval.

^a^Reference: temperature ≥20°C.

^b^Reference: day without precipitation.

^c^Reference: day without snow.

Light precipitation was defined as less than 3.5 mm (the median), and heavy precipitation was defined as 3.5 mm or more.

Table 3 shows that the effects of snow precipitation on road traffic injuries depending on temperature. The effect of snow on road traffic injuries was increased by 15% when the mean daily temperature was below 0°C. When the temperature fell below freezing, the risk of all types of road traffic injuries, except car-to-pedestrian accidents, increased in the presence of snow. Additionally, the combined effect of snow precipitation and cold temperatures was especially pronounced with regard to mild injuries, car-to-car accidents, accidents involving male drivers, and accidents involving drivers younger than 65 years. Injuries from car-to-car accidents increased by 18.6% (95% CI, 16.8%–20.5%) when the mean temperature fell below −5°C with snow compared with those on dry days above 0°.

**Table 3.** Interactive effect of temperature and snow on road traffic injuries, 2007 to 2011

	No snow		Snow	
	
	% change	95% CI	% change	95% CI
All road traffic injuries				
<−5°C	2.81	1.43, 4.21	14.8	13.2, 16.5
−5–0°C	−0.10	−1.11, 0.91	15.1	13.7, 16.6
>0°C	Ref	—	6.56	5.04, 8.09
Severe road traffic injuries^a^				
<−5°C	1.92	−2.32, 6.34	1.08	−3.42, 5.79
−5–0°C	0.28	−2.80, 3.45	6.76	2.55, 11.1
>0°C	Ref	—	4.32	−0.25, 9.11
Mild road traffic injuries^a^				
<−5°C	2.94	1.49, 4.42	16.4	14.7, 18.1
−5–0°C	−0.13	−1.19, 0.94	16.1	14.6, 17.6
>0°C	Ref	—	6.73	5.13, 8.36
Car-to-car accidents				
<−5°C	3.88	2.37, 5.41	18.6	16.8, 20.5
−5–0°C	0.69	−0.41, 1.80	17.6	16.0, 19.2
>0°C	Ref	—	5.90	4.26, 7.58
Car-to-pedestrian accidents				
<−5°C	−3.23	−7.08, 0.78	−7.77	−11.8, −3.61
−5–0°C	−4.49	−7.29, −1.61	−2.25	−5.98, 1.63
>0°C	Ref	—	11.6	7.20, 16.2
Male drivers				
<−5°C	2.64	1.12, 4.18	17.3	15.5, 19.2
−5–0°C	−0.37	−1.47, 0.75	16.4	14.8, 18.0
>0°C	Ref	—	7.11	5.43, 8.81
Female drivers				
<−5°C	3.53	0.25, 6.91	3.09	−0.41, 6.72
−5–0°C	0.94	−1.45, 3.38	8.85	5.59, 12.2
>0°C	Ref	—	3.80	0.30, 7.43
Young drivers^b^				
<−5°C	3.01	1.60, 4.43	15.2	13.6, 16.9
−5–0°C	−0.11	−1.13, 0.92	15.6	14.1, 17.0
>0°C	Ref	—	6.79	5.25, 8.35
Elderly drivers^b^				
<−5°C	−3.48	−10.6, 4.16	2.60	−5.05, 10.9
−5–0°C	−0.51	−5.89, 5.17	0.75	−6.25, 8.28
>0°C	Ref	—	−2.36	−10.2, 6.10

CI, confidence interval.

^a^Severe injuries required more than 3 weeks of medical treatment, and mild injuries required less than 3 weeks of treatment.

^b^An accident was categorized as involving a young driver when the responsible party was younger than 65 years of age and as involving an elderly driver when the responsible party was aged 65 years or older.

Overall, heavy rain precipitation was related to an increase in road traffic injuries when the temperature was moderate (Table 4). Compared with hot and dry days, injuries increased by 12.2% on days with heavy rain precipitation and moderate temperatures (between 0°C and 20°C). On such days, the risk increased by 8.45% and 6.79% over the values on days with a mean temperature below 0°C and above 20°C, respectively. An interactive effect of rain precipitation and temperature was also detected in our analysis according to the road traffic injury subtype. On days with heavy rain precipitation, injuries from car-to-car accidents increased by 14.4% (95% CI, 13.2%–15.6%) when the temperature was 0–20°C compared with hot and dry days. However, the risk of road traffic injuries on days with rain precipitation was reduced in some groups when the temperature was below freezing. Injuries related to car-to-pedestrian accidents and elderly drivers were reduced by 10.2% and 22.6%, respectively, on such days.

**Table 4.** Effect of the relationship between temperature and amount of rain precipitation on road traffic injuries, 2007 to 2011

	No precipitation		Light precipitation		Heavy precipitation	
		
	% change	95% CI	% change	95% CI	% change	95% CI
All road traffic injuries						
<0°C	1.31	0.12, 2.51	−1.66	−3.34, 0.05	8.45	5.48, 11.5
0–20°C	−2.10	−2.92, −1.28	0.32	−0.68, 1.33	12.2	11.1, 13.2
>20°C	Ref	—	−3.08	−3.92, −2.23	6.79	5.99, 7.61
Severe road traffic injuries^a^						
<0°C	−0.06	−3.62, 3.63	−4.88	−10.0, 0.55	−3.53	−12.1, 5.91
0–20°C	−2.58	−5.03, −0.07	−0.02	−3.04, 3.09	8.11	5.05, 11.3
>20°C	Ref	—	−1.68	−4.30, 1.01	6.36	3.87, 8.91
Mild road traffic injuries^a^						
<0°C	1.54	0.29, 2.52	−1.24	−3.01, 0.57	9.81	6.67, 13.0
0–20°C	−2.00	−2.86, −1.13	0.41	−0.64, 1.48	12.7	11.6, 13.8
>20°C	Ref	—	−3.26	−4.14, −2.36	6.84	5.99, 7.70
Car-to-car accidents						
<0°C	3.25	1.93, 4.58	−0.70	−2.54, 1.17	10.3	7.03, 13.7
0–20°C	−1.77	−2.67, −0.86	1.09	−0.04, 2.20	14.4	13.2, 15.6
>20°C	Ref	—	−3.25	−4.16, −2.33	7.86	6.97, 8.76
Car-to-pedestrian accidents						
<0°C	−10.1	−13.2, −6.92	−9.13	−13.8, −4.23	−10.2	−17.5, −2.16
0–20°C	−4.87	−7.15, −2.54	−2.87	−5.63, −0.04	1.13	−1.60, 3.93
>20°C	Ref	—	−1.91	−4.38, 0.62	1.00	−1.28, 3.33
Male drivers						
<0°C	1.80	0.49, 3.13	−1.48	−3.32, 0.41	10.6	7.26, 13.9
0–20°C	−1.62	−2.53, −0.71	0.93	−0.17, 2.05	13.4	12.3, 14.6
>20°C	Ref	—	−3.28	−4.21, −2.35	7.79	6.89, 8.69
Female drivers						
<0°C	−0.76	−3.49, 2.04	−2.35	−6.34, 1.81	−0.86	−7.46, 6.20
0–20°C	−4.00	−5.87, −2.10	−2.18	−4.45, 0.14	6.96	4.62, 9.34
>20°C	Ref	—	−2.23	−4.20, −0.22	2.20	0.37, 4.07
Young drivers^b^						
<0°C	1.31	0.10, 2.52	−1.40	−3.11, 0.34	9.54	6.51, 12.7
0–20°C	−2.17	−3.00, −1.33	0.56	−0.46, 1.59	12.5	11.5, 13.6
>20°C	Ref	—	−3.30	−4.16, −2.44	7.17	6.35, 8.00
Elderly drivers^b^						
<0°C	1.99	−4.41, 8.82	−8.84	−1.75, 0.70	−22.6	−35.2, −7.50
0–20°C	0.88	−3.60, 5.56	−5.44	−10.5, −0.09	3.14	−2.08, 8.64
>20°C	Ref	—	3.50	−1.29, 8.52	−4.35	−8.39, −0.13

CI, confidence interval.

^a^Severe injuries required more than 3 weeks of medical treatment, and mild injuries required less than 3 weeks of treatment.

^b^An accident was categorized as involving a young driver when the responsible party was younger than 65 years of age and as involving an elderly driver when the responsible party was aged 65 years and older.

**Figure 2.**
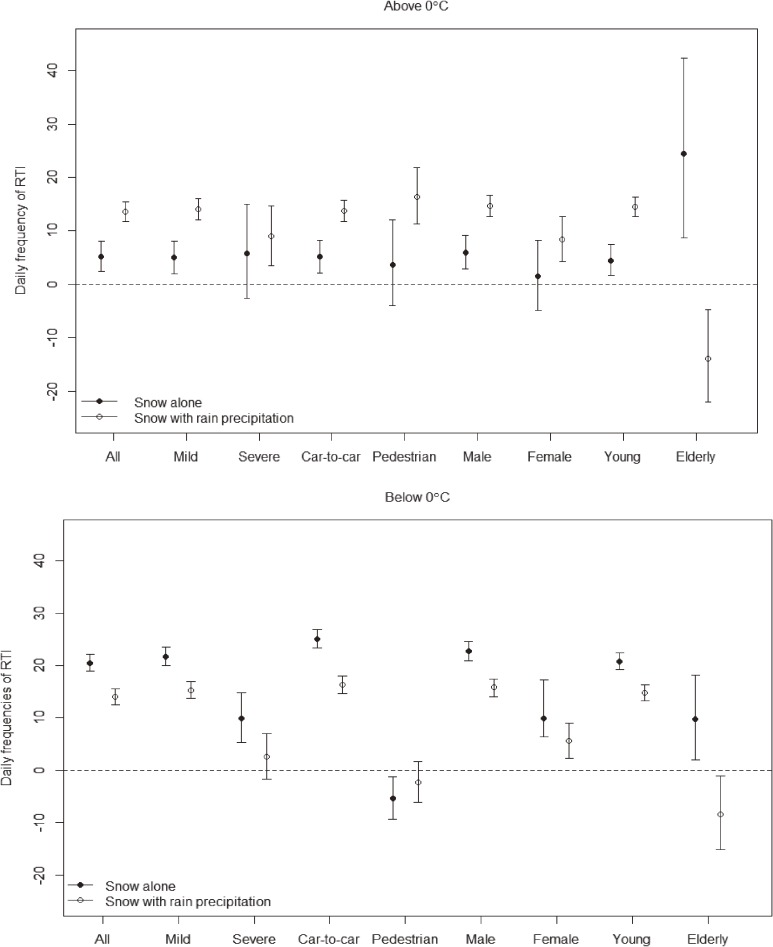
Risk estimates for snow alone and snow combined with rain precipitation on road traffic injuries by temperature, 2007–2011. “All” means all road traffic injuries (RTI); “Severe” means severe RTI which required ≥3 weeks of medical treatment, while “Mild” means mild RTI which required less than 3 weeks of treatment; “Car-to-car” is RTI from car-to-car accidents, while “Pedestrian” is RTI from car-to-pedestrian accidents; “Male” means RTI induced by male drivers, while “Female” means RTI induced by female drivers; “Young” is RTI induced by drivers younger than 65 years of age, while “Elderly” is RTI induced by the drivers aged 65 years and older.

Rain precipitation fell on existing snow on 64 days (3.6% of the study period) (Figure 2). Overall, rain precipitation on existing snow was associated with more road traffic injuries than was snow alone when the temperature was above 0°C. Days with a mean temperature above 0°C during which rain precipitation fell on existing snow were characterized by an increase in the risk of road traffic injuries by 13.6% (95% CI, 11.7%–15.4%), which was higher than the 5.20% increase (95% CI, 2.39%–8.08%) when only snow fell. In contrast, rain precipitation on existing snow was associated with a lower risk of road traffic injuries than was snow alone when the mean daily temperature was below 0°C. When rain precipitation fell on snow, the increase in the risk of road traffic injuries was 14.0% on days with a mean temperature below 0°C (95% CI, 12.5%–15.5%), which was lower than the 20.5% (95% CI, 18.9%–22.2%) increase in the risk of injuries when only snow was observed on the roads. The risk of injuries from car-to-pedestrian accidents did not increase when the mean temperature was below 0°C; indeed, the risk was −5.44% for snow and −2.36% for rain precipitation on existing snow. Moreover, injuries caused by elderly drivers were reduced when rain precipitation fell on existing snow, regardless of the temperature.

## DISCUSSION

The risk of road traffic injuries in rain or snow precipitation differs depending on the temperature. Snow was associated with the highest risk of injuries from traffic accidents under freezing temperature conditions. In contrast, rain precipitation was related to the highest risk of road traffic injuries in moderate temperatures. The risk of road traffic injuries when rain precipitation fell on existing snow differed from the effect of snow alone according to temperature. Moreover, these differences were observed among almost all traffic accident subtypes.

Adverse weather conditions contributed to an increased risk of road traffic injuries. Although a few studies have reported a negative relationship between traffic accidents and snow,^20^^,^^21^ most epidemiological studies have found that the risk of road traffic injuries increases when it snows. Indeed, one meta-analysis reported that road traffic injuries increased by 75% (95% CI, 54%–96%) when it snowed,^15^ and a recent study conducted in Canada found that injuries from traffic accidents increased by 47% on snowy days.^22^ Our result is consistent with these studies, and the risk of road traffic injuries in the present study increased by 11% on snow. The risk increase in our research is lower than in previous reports. The difference could depend on factors such as meteorological characteristics and road conditions of the study area, as well as definitions in the reports. For example, it snows less heavily in Seoul than in any of the six cities in the Canadian study, so the effect of a ‘snowy day’ could be different when snowy day is defined differently based on the amount of snow.^22^

Additionally, the effects of snow on traffic accidents have decreased with time. A previous review reported that the risk increases for snow-related traffic accidents during 1950–1979, 1980–1989, and 1990–2005 were 113%, 71%, and 47%, respectively.^15^ Conversely, most previous studies have suggested that rain precipitation is associated with a higher risk of traffic accidents.^11^^,^^23^ During rain precipitation, road traffic injuries has been reported to increase by 21%, 50%, and 42% in the United States, Canada, and the United Kingdom, respectively.^15^ Similarly, we also found a possible association between rain precipitation and short-term risk for road traffic injuries. Indeed, the risk of road traffic injuries increased by 10.5% during rain precipitation of more than 3.5 mm. This increase in the risk of road traffic injuries differed from those reported previously. Similar to snow, the differences could result from variations in study periods, regions, and definitions of exposure and outcomes.^15^^,^^16^^,^^22^^,^^24^ In a study in which the definitions were established according to duration and amount of rainfall, the risk associated with rain was greater than that in studies where rain was defined as the existence of rain.^15^ They indicated only modest sensitivity when case events of rainy day were defined as combinations of rainfall amounts of 0 mm, 0.2 mm and 0.4 mm with rainfall duration of 0 hours and 3 hours.

The separate and interactive effects of temperature on road traffic injuries have received relatively little research attention. A few studies have shown an indirect and inconsistent relationship between temperature and injuries. Several epidemiological studies have suggested that increased temperature was related to the increased use of emergency medical services or increased admissions to trauma centers.^25^^–^^27^ In contrast, some evidence showed that moderate rather than hot temperatures were related to the highest rate of injuries. A previous study suggested that the peak incidence of trauma admissions occurred in May in the United Kingdom.^28^ Another study suggested that traumatic injuries, including road traffic injuries, were non-linearly associated with peak temperatures of about 20°C.^29^

Road traffic injuries on snow may be attributable to the slipperiness of wet pavement.^30^^,^^31^ Physicists and automobile engineers measure the slipperiness of wet pavement by the friction coefficient. Decreased friction coefficients may be related to increased braking distances and loss of control by drivers. Temperature may interact with wet road conditions, which interfere with the contact between the tires and the road surface, decreasing the friction coefficient. Previous research found that compacted and uncompacted snow had low friction coefficients (0.24–0.37 and 0.15–0.42, respectively) compared with dry asphalt (0.59–0.72).^32^ In contrast, black ice and ice had friction coefficients of 0.12–0.26 and 0.054–0.019, respectively, which are lower than that of snow. These data are consistent with our finding that snow can change into black ice or ice, yielding a higher risk of road traffic injuries when the temperature is below freezing. Additionally, a high risk of road traffic injuries could be related to cold temperature because of the longer night and poorer visibility in winter.^33^

In terms of rain precipitation, moderate temperature may be associated with more road traffic injuries because people participate in more outdoor activities in moderate temperatures. In the GAM model, we tried to adjust for seasonal variation in traffic volume using a smoothing spline for date. However, the effect of traffic volume may have been under-adjusted, and thus some effect might have remained. Spring (March to May) and two months during autumn (October and November) correspond with moderate temperature, defined conditionally from 0°C to 20°C, according to the annual climatological report in Seoul, Korea.^34^ When we compared monthly average traffic volume in 10 downtown spots, spring and autumn showed more traffic volume than summer and winter. The respective ratios of monthly average weekday traffic to annual average weekday traffic ranged from 1.01 to 1.03 in spring and autumn to 0.94 to 0.99 in summer and winter.^35^ These were also observed on the bridges and highways in Seoul. Therefore, moderate temperature in times of rain precipitation might be related to more injuries due to heavier traffic.

We found consistency in the interactive effect of temperature and wet conditions among the different subtypes of road traffic injuries. When rain precipitation occurred in the context of moderate temperatures, the risk of road traffic injuries increased regardless of the sex and age of drivers, the severity of injuries, or the type of traffic accidents. When the temperature dropped below freezing, the risk of all types of road traffic injuries increased among all subgroups in the snow, with the exception of injuries from car-to-pedestrian accidents, which decreased. Consistencies were also found among the subgroups under conditions involving rain precipitation on existing snow. Differences among subgroups in the extent to which risks were increased might be due to differences in attitudes towards risk avoidance, the willingness to use public transportation, and safe driving to reduce speed and maintain a safe distance from other cars.^12^^,^^17^ The effect of inclement weather on road traffic injuries to pedestrians was much lower than on car-to-car accidents in this study. One hypothesis explaining this relationship is that pedestrians are less likely to jaywalk in inclement weather, while drivers still violate traffic signals and do not keep a safe distance. However, little is known about drivers and pedestrian behaviors in inclement weather.^36^

This study suggests an interactive effect of inclement weather and temperature on road traffic injuries. There could be some discrepancy between number of traffic accidents and road traffic injuries. However, risk increases for road traffic injuries were quite similar to those of traffic accidents, as indicated by their high correlation (*r* = 0.91). When it snowed, the risk of traffic accidents increased by 12.4%, 11.8%, and 7.34% over the values on days with a mean temperature below −5°C, −5°C to 0°C, and above 0°C, respectively. Corresponding increases in risk of road traffic injuries were 14.8%, 15.1%, and 6.56%, respectively. Regarding the mixed effect of rain and snow precipitation, the pattern of risk increase in traffic accidents was comparable to that of road traffic injuries (Figure 2). Risk of traffic accidents showed a greater increase in conditions of rain precipitation on existing snow (13.1%) than snow alone (6.86%) when it was above 0°C, while the risk of the latter (16.9%) was higher than the former (10.9%) below 0°C. The purpose of this study was to evaluate the influence of meteorological factors not on the number of traffic accidents, but from the point of view of human health. Thus, injuries from traffic accidents should be considered the outcome of the current research.

A strength of the current research is its use of accurate information about the risk of road traffic injuries to examine the interactive effects of temperature under conditions of rain and/or snow precipitation. Second, the short-term effects of rain precipitation, snow precipitation, and temperature were evaluated after seasonality and long-term trends were adjusted by the spline function of GAM. Additionally, we used an integrated database that included not only severe but also minor injuries, which would be excluded from records of trauma centers or emergency medical services.

One limitation of the study was that the types of and body regions affected by road traffic injuries could not be analyzed because the data did not include diagnostic codes. Additionally, the severity of injuries was defined in terms of the duration of treatment, which made it difficult to compare our results with those of previous studies. Finally, data on daily traffic volumes were not available for the study period, so the effects of increased traffic volume, which are possible in inclement weather, could be not considered. However, whether the increase in road traffic injuries during times of rain and snow precipitation are due to increased traffic volume remains unclear. Previous researchers have suggested a negative relationship between rainfall^37^ and traffic volume, and that the risk of normalized traffic accidents in rain increased by 2.4% when adjusted for traffic volume.^38^

### Conclusions

This study revealed that the effect of adverse weather conditions on road traffic injuries differs depending on the mean temperature of the day. A greater number of road traffic injuries were associated with rain precipitation when the temperature was moderate and with snow when it was freezing. The interactive effect of temperature and wet road conditions was consistent across traffic accident subtypes. These results can be used to prepare information regarding the risk of road traffic injuries for dissemination to the public.
